# Glutamine, fatty liver disease and aging

**DOI:** 10.18632/aging.202666

**Published:** 2021-02-11

**Authors:** Jorge Simón, María Luz Martínez-Chantar, Teresa C. Delgado

**Affiliations:** 1 Liver Disease Laboratory, Center for Cooperative Research in Biosciences (CIC bioGUNE), Basque Research and Technology Alliance (BRTA), Bizkaia Technology Park, Derio, Bizkaia 48160, Spain; 2 Centro de Investigación Biomédica en Red de Enfermedades Hepáticas y Digestivas (CIBERehd), Derio, Bizkaia 48160, Spain

**Keywords:** NAFLD, lutaminase, glutaminolysis, hepatocytes, hepatic stellate cells

Non-alcoholic fatty liver disease (NAFLD), characterized by the accumulation of hepatic fat due to non-alcoholic and non-viral causes, is the commonest form of chronic liver disease in western countries affecting over 25% of the worldwide population [1]. In spite of the epidemic proportions and gloomy future panorama of NAFLD, the causes and mechanisms underlying its progression are not completely understood and besides lifestyle interventions and weight loss by bariatric surgery, there are currently no approved therapies towards NAFLD.

Very recently, deregulated metabolism of the amino acid glutamine has been implicated in non-alcoholic steatohepatitis (NASH), a condition localized in the most harmful spectrum of NAFLD and characterized by increased necro-inflammatory response in a steatotic background. Indeed, our group has shown that the high activity isoform of the enzyme glutaminase, the glutaminase kidney isoform (GLS), an enzyme that accounts for the conversion of glutamine to glutamate and ammonia catalyzing the first step of glutaminolysis and usually minority in the healthy liver, is aberrant in liver biopsies of pre-clinical mouse models of early NASH as well as in NASH patients [2]. Similar findings from other authors have shown that glutaminolysis is a potential diagnostic marker during advanced NASH fibrosis [3], being a regulator of myofibroblasts hepatic stellate cell (HSC) accumulation, the main fibrogenic cell type [4]. To date, the mechanisms underlying the upregulation of GLS in NASH, that appears to occur at the transcriptional level, remain to be unsolved and further studies are necessary to unravel potential regulators of GLS1 in NASH. Hypothetically, GLS promoter methylation could be relevant, similar to what occurs in Hepatocellular Carcinoma (HCC), as well as microRNAs-mediated upregulation of gene expression.

Importantly, we have shown that GLS silencing and inhibition *in vivo* by using molecular approaches specifically targeting the liver can attenuate NASH both by reducing the accumulation of lipids and reactive oxygen species (ROS) in pre-clinical animal models of methionine and/or choline restricted diets-induced NASH [2]. In addition, other authors have shown that GLS pharmacological inhibition can reduce the activation of HSC in mouse models of carbon tetrachloride (CCl_4_)-induced acute liver fibrosis [4]. Of highlight, numerous medicinal chemistry studies are currently aimed at the design of novel and potent inhibitors for GLS, mainly focusing on its utilization in oncotherapy. Thereby, by combining its anti-steatotic and anti-fibrotic properties, GLS1 targeting appears to be an effective and safe candidate target for the pharmacological treatment of NASH.

Aging is the most common cause for the progression of NAFLD [5] and is intrinsically associated with changes in systemic metabolism, including changes in the amino acids, lipids, sugar, and nucleotide metabolism [6]. Of interest, an aging-associated metabolomic signature in rodents highlights that there is a significant negative correlation of the serum amino acid glutamine in wild-type samples with age [7]. Lower serum glutamine levels observed in aged mice overlap with the reduced amount of this metabolite observed in mouse models of advanced liver fibrosis [3]. One hand, considering that NAFLD is very prevalent in the elderly population, metabolomics-based studies addressing the real impact of NAFLD or even more advanced NASH in the reduced serum glutamine levels of elderly patients or pre-clinical animal models should be considered. And on the other hand, the real impact of the expression of GLS in elderly livers should be investigated. Alterations in hepatic glutamine catabolism with aging could potentially indicate that the low concentrations of serum glutamine in elderly are not only due to sarcopenia, age-related muscle loss and strength waste, but also to the increased hepatic utilization of glutamine. Finally, the concept that glutamine usage in the liver could be altered in elderly is relevant for the safety of oral use of glutamine supplements, that are seldom indicated for the treatment of some type of disease or recommended in sports nutrition. Indeed, potential differences in the hepatic glutamine metabolism with aging could partially explain the small increases in serum ammonia observed after oral L-glutamine nutraceutical doses in elderly patients [8] and under these circumstances a rigorous control of both renal and hepatic function are imposed.

**Figure 1:**
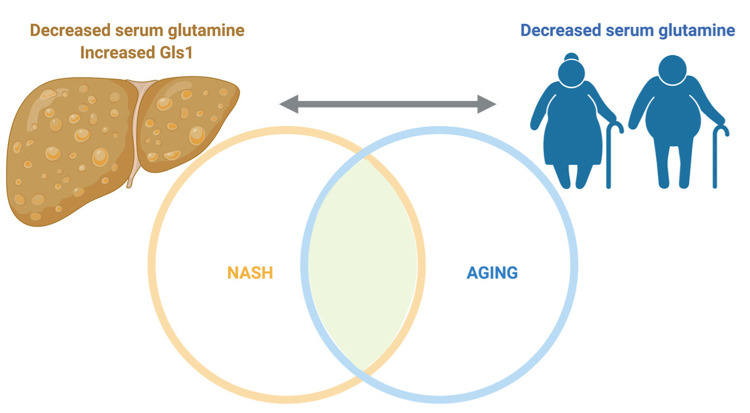
**Glutamine, fatty liver disease and aging.** Non-alcoholic steatosis is characterized by decreased serum glutamine and increased expression of hepatic glutaminase. Aging, the most common cause of non-alcoholic fatty liver (NAFLD) and NASH is also characterized by low serum glutamine levels. The relationship between serum glutamine, aging and fatty liver remains to be unraveled. Figure created with BioRender.com.

In summary, glutamine catabolism plays a role in the regulation of non-alcoholic fatty liver disease, an aging-related disease, whereas serum glutamine is altered in aging (Figure 1). Further studies are necessary to clarify if changes if serum glutamine are related to aging or derived from the highly prevalent condition of NAFLD in aged patients.
